# A Descriptive Study on Opioid Misuse Prevalence and Office-Based Buprenorphine Access in Ohio Prior to the Removal of the Drug Addiction Treatment Act of 2000 Waiver

**DOI:** 10.7759/cureus.36903

**Published:** 2023-03-30

**Authors:** Erin R McKnight, Qianyu Dong, Daniel L Brook, Staci A Hepler, David M Kline, Andrea E Bonny

**Affiliations:** 1 Department of Pediatrics, Division of Adolescent Medicine, The Ohio State University, Nationwide Children's Hospital, Columbus, USA; 2 Department of Statistical Sciences, Wake Forest University, Winston-Salem, USA; 3 Division of Epidemiology, The Ohio State University College of Public Health, Columbus, USA; 4 Department of Biostatistics and Data Science, Wake Forest University, Winston-Salem, USA

**Keywords:** access, prevalence, prescriptions, buprenorphine, opioid overdose

## Abstract

Background

Medications for the treatment of opioid use disorder (MOUD) are effective evidence-based strategies to reduce opioid overdose deaths. Strategies to optimize MOUD availability and uptake are needed.

Objective

We aim to describe the spatial relationship between the estimated prevalence of opioid misuse and office-based buprenorphine access in the state of Ohio prior to the removal of the Drug Addiction Treatment Act of 2000 (DATA 2000) waiver requirement.

Methods

We conducted a descriptive ecological study of county-level (N=88) opioid misuse prevalence and office-based buprenorphine prescribing access in Ohio in 2018. Counties were categorized into urban (with and without a major metropolitan area) and rural. The county-level prevalence estimates of opioid misuse per 100,000 were derived from integrated abundance modeling. Utilizing data from the Ohio Department of Mental Health and Addiction Services, as well as the state’s Physician Drug Monitoring Program (PDMP), buprenorphine access per 100,000 was estimated by the number of patients in each county that could be served by office-based buprenorphine (prescribing capacity) and the number of patients served by office-based buprenorphine (prescribing frequency) for opioid use disorder. The ratios of opioid misuse prevalence to both prescribing capacity and frequency were calculated by county and mapped.

Results

Less than half of the 1,828 waivered providers in the state of Ohio in 2018 were prescribing buprenorphine, and 25% of counties had no buprenorphine access. The median estimated opioid misuse prevalence and buprenorphine prescribing capacity per 100,000 were highest in urban counties, particularly those with a major metropolitan area. Although the median estimated opioid misuse prevalence was lower in rural counties, all counties in the highest quartile of estimated misuse prevalence were rural. In addition, the median buprenorphine prescribing frequency was highest in rural counties.

While the ratio of opioid misuse prevalence to buprenorphine prescribing capacity was lowest in urban counties, the ratio of opioid misuse prevalence to buprenorphine prescribing frequency was lowest in rural counties. Opioid misuse prevalence and buprenorphine prescribing frequency demonstrated similar spatial patterns, with highest levels in the southern and eastern portions of the state, while office-based buprenorphine prescribing capacity did not.

Conclusion

Urban counties had higher buprenorphine capacity relative to their burden of opioid misuse; however, access was limited by buprenorphine prescribing frequency. In contrast, in rural counties, a minimal gap was evident between prescribing capacity and frequency, suggesting that buprenorphine prescribing capacity was the major factor limiting access. While the recent deregulation of buprenorphine prescribing should help improve buprenorphine access, future research should investigate whether deregulation similarly impacts buprenorphine prescribing capacity and buprenorphine prescribing frequency.

## Introduction

Since the early 2000s, the United States (US) has seen an alarming increase in opioid-related mortality nationwide. While there were some decreases in overdose death rates in 2018 and 2019, the onset of the COVID-19 pandemic led to a worsening in the opioid overdose crisis. More than 100,000 overdose deaths were recorded in 2021, representing the highest annual overdose death count in US history, and about two-thirds of these deaths involved fentanyl or another synthetic opioid [1]. This unprecedented overdose death rate requires immediate, data-informed, targeted action.

The overdose crisis is particularly severe in Ohio. Ohio’s age-adjusted annual death rate per 100,000, due to unintentional drug poisonings, increased from 8.9 in 2005 to 44.1 in 2017. Like national patterns, this increase was largely driven by opioid-involved overdoses [2,3]. While Ohio’s overdose death rate decreased by 22.5% in 2018, the age-adjusted opioid overdose death rate was 29.6 per 100,000 in Ohio compared to 20.7 per 100,000 nationally [2,4]. Following the declaration of the COVID-19 national emergency in March 2020, overdose deaths rose sharply in Ohio. Compared to the same month in 2019, there was a 76.8% increase in overdose deaths in May 2020 [5].

Medications for the treatment of opioid use disorder (MOUD) are effective evidence-based strategies to reduce opioid overdose deaths and have been endorsed as a primary strategy for addressing the opioid crisis by the US Department of Health and Human Services’ Opioid Initiative [6,7]. MOUD includes two classes of medications, opioid agonists and antagonists. Methadone, a full opioid agonist, must be administered through a Substance Abuse and Mental Health Services Administration (SAMHSA)-certified opioid treatment program (OTP). Buprenorphine, a partial opioid agonist, may be prescribed or administered in OTPs but can also be prescribed in less regulated office-based opioid treatment (OBOT) settings. Until recently, buprenorphine prescribing in OBOTs was limited to providers who received additional training and subsequently registered for a waiver to prescribe or dispense buprenorphine under the Drug Addiction Treatment Act of 2000 (DATA 2000) [8]. Providers authorized under the DATA 2000 waiver could prescribe to a maximum of 30-275 patients per provider [9]. In January 2023, the practice guidelines for the administration of buprenorphine were adjusted, eliminating the waiver requirement and no longer limiting the number of patients a prescriber can treat [10]. MOUD also includes naltrexone, an opioid antagonist that has less data supporting its effectiveness in reducing overdose and drug use-related acute care visits compared to agonist therapies [11]. While methadone may be best for supporting treatment retention [12], particularly for patients on fentanyl, buprenorphine is uniquely positioned to have broader availability relative to methadone given the regulations around prescribing the latter.

National data finds that access to MOUD is dependent on geography and treatment referral streams [13]. Strategies to optimize the availability and uptake of MOUD are needed to ensure that this life-saving treatment is reaching the populations most in need. The current study’s objective was to describe the spatial relationship between the estimated prevalence of opioid misuse and office-based buprenorphine access in the state of Ohio prior to the removal of the DATA 2000 waiver requirement.

## Materials and methods

We conducted a descriptive ecological study of the estimated prevalence of opioid misuse and office-based buprenorphine access for all 88 counties in Ohio in 2018.

Opioid misuse prevalence

Surveillance of behaviorally linked conditions, such as substance use, is challenging. These conditions tend to vary at the local level, and formal diagnosis of a Diagnostic and Statistical Manual of Mental Disorders, Fifth Edition (DSM-5) substance use disorder requires accessing specific healthcare. Given multiple barriers to accessing care, which can vary by location and across time, many individuals with problematic opioid use never receive a formal opioid use disorder diagnosis. To more accurately represent the burden of problematic opioid use, county-level opioid misuse was estimated using an integrated abundance model described by Hepler et al. [14]. Utilizing a Bayesian hierarchical spatiotemporal abundance model, Hepler et al. [14] integrated county-level data on opioid-related outcomes (i.e., opioid overdose deaths and opioid use disorder treatment admissions) with state-level survey estimates on the prevalence of opioid misuse [15,16] to estimate the latent county-level prevalence and counts of people who misused opioids in 2018. Direct estimates of opioid misuse at the county level are lacking, and considering just a single opioid-related outcome, such as overdose deaths, can be misleading since unobserved opioid misuse may manifest differently across geographical regions based on elements such as supply and available resources. Thus, no single outcome can accurately reflect the underlying geographical distribution or the severity of opioid misuse. The integrated abundance model overcomes this limitation by integrating multiple outcomes including treatment admissions for opioid use disorder and opioid-related overdose deaths. A detailed description of this model has been previously published [14]. While our estimate may be an overestimation of individuals needing treatment, utilizing only individuals with a diagnosis of OUD would be an underestimation.

Office-based buprenorphine access

Office-based buprenorphine access included both buprenorphine prescribing capacity and frequency.

Buprenorphine Prescribing Capacity

The Ohio Department of Mental Health and Addiction Services (OMHAS) provided information on the number of physicians (MD or DO) with an active DATA 2000 waiver certification in quarter 1 (March 31) of 2018. We calculated the prevalence of patients per 100,000 population in each county who could have potentially been treated by a prescriber with a waiver (office-based buprenorphine prescribing capacity) from January to March 2018. This outcome was generated by aggregating the legal limit of all waivered providers in a county. This measure reflects a “best case scenario” by county of available office-based buprenorphine.

Buprenorphine Prescribing Frequency

The state’s Physician Drug Monitoring Program (PDMP) was accessed to determine the actual number of patients who received a prescription for buprenorphine for opioid use disorder in 2018. We calculated the average number of patients per 100,000 population in each county receiving an office-based buprenorphine prescription (office-based buprenorphine prescribing frequency) in each quarter of 2018. This measure reflects the “real-life availability” by county of office-based buprenorphine.

Opioid treatment programs

OTP locations were obtained from the SAMHSA OTP directory (https://dpt2.samhsa.gov/treatment/). This directory lists the first full certification date for each program that was utilized to identify which of Ohio’s OTPs were certified in 2018.

County designation

Rural and urban county categorizations were gathered from the Ohio Urban County Directory (https://urban-extension.cfaes.ohio-state.edu/positioning/ohio-urban-county-directory). Urban counties were then further delineated into those with and without major metropolitan areas (Columbus, Cleveland, Cincinnati, Toledo, Akron, and Dayton).

Statistical analyses

To describe the spatial distribution of opioid misuse and buprenorphine access, we mapped the county-level rates per 100,000 residents of estimated opioid misuse, buprenorphine prescribing capacity, and buprenorphine prescribing frequency. The 25th, 50th, and 75th percentiles were calculated for opioid misuse prevalence, buprenorphine capacity, and buprenorphine frequency. Within the bottom quartile, we separated out counties with no buprenorphine capacity to better highlight these areas. We overlaid the locations of OTPs to illustrate the overlap between access to OTPs and buprenorphine.

The estimated rates of opioid misuse were plotted against buprenorphine prescribing capacity and frequency. We also computed ratios of the estimated counts of misuse relative to the prescribing capacity and prescribing frequency. Ratios were not generated for counties with no buprenorphine capacity as zero in the denominator made these undefined. The following cutoffs were utilized to categorize the ratios of estimated misuse counts and buprenorphine capacity: 0.0-1.0, 1.0-5.0, 5.0-10.0, 10.0-25.0, and 25.0 or greater. The cutoffs for the estimated misuse counts and buprenorphine frequency were as follows: 1.0-5.0, 5.0-10.0, 10.0-25.0, and 25.0 or greater. Ratios were then mapped to characterize spatial trends in opioid misuse relative to access to buprenorphine. Counties where estimated opioid misuse prevalence was above the state median and access to buprenorphine was below the state median were highlighted on the maps.

## Results

All 88 Ohio counties were included in the analyses. In total, 1,828 physicians were certified to prescribe office-based buprenorphine, with 859 (47%) actively prescribing buprenorphine. Fifteen (17%) counties had no physicians certified to prescribe office-based buprenorphine, and an additional seven (8%) counties had physicians who had a certification to prescribe office-based buprenorphine but did not actually prescribe office-based buprenorphine in 2018.

Among the 88 Ohio counties, 15 were categorized as urban and 73 as rural. Six of the urban counties contained a major metropolitan area. The median estimated opioid misuse prevalence and buprenorphine prescribing capacity per 100,000 were highest in the six urban counties that contained a major metropolitan area (Table 1). Although the median estimated opioid misuse prevalence was lower in rural counties, all counties in the highest quartile of estimated misuse prevalence were rural. In addition, the median buprenorphine prescribing frequency was highest in the 73 rural counties.

**Table 1:** Opioid misuse and buprenorphine access characteristics by Ohio counties IQR: interquartile range

Variable, median (IQR)	All (N=88)	Urban (n=15)	Urban with major metropolitan (n=6)	Urban without major metropolitan (n=9)	Rural (n=73)
Opioid misuse prevalence/100,000	3749.36 (3211.78-4694.91)	3744.63 (3522.56-4404.90)	3955.02 (3734.25-4586.96)	3662.27 (2969.64-4012.90)	3754.09 (3188.15-4694.53)
Buprenorphine prescribing capacity/100,000	676.94 (236.47-1179.76)	1359.69 (855.20-2048.43)	2048.43 (1520.14-2300.21)	983.32 (726.64-1359.69)	518.72 (178.87-1034.46)
Buprenorphine prescribing frequency/100,000	473.63 (304.50-761.69)	352.22 (312.44-502.41)	408.35 (339.54-470.81)	332.84 (303.44-531.90)	501.57 (304.85-823.93)
Opioid misuse prevalence/buprenorphine prescribing capacity	5.84 (3.66-15.80)	2.88 (1.64-4.44)	2.53 (1.77-2.86)	4.09 (1.66-4.93)	6.83 (4.45-22.43)
Opioid misuse prevalence/buprenorphine prescribing frequency	8.35 (5.72-10.92)	9.89 (8.49-10.57)	10.12 (9.05-10.73)	8.52 (7.65-10.27)	7.55 (5.24-11.33)

The median ratio of opioid misuse prevalence to buprenorphine prescribing capacity (Table 1) was lowest in urban counties that contained major metropolitan areas (urban counties with major metropolitan area: 2.53, urban counties without major metropolitan area: 4.09, rural counties: 6.83), indicating that rural counties had a higher number of estimated individuals needing treatment relative to the number of patients that could be treated. In contrast, the ratio of opioid misuse prevalence to buprenorphine prescribing frequency was lowest in the rural counties (urban counties with major metropolitan area: 10.12, urban counties without major metropolitan area: 8.52, rural counties: 7.55), indicating that rural counties had the lowest number of estimated individuals needing treatment relative to the number of patients receiving treatment.

**Figure 1:**
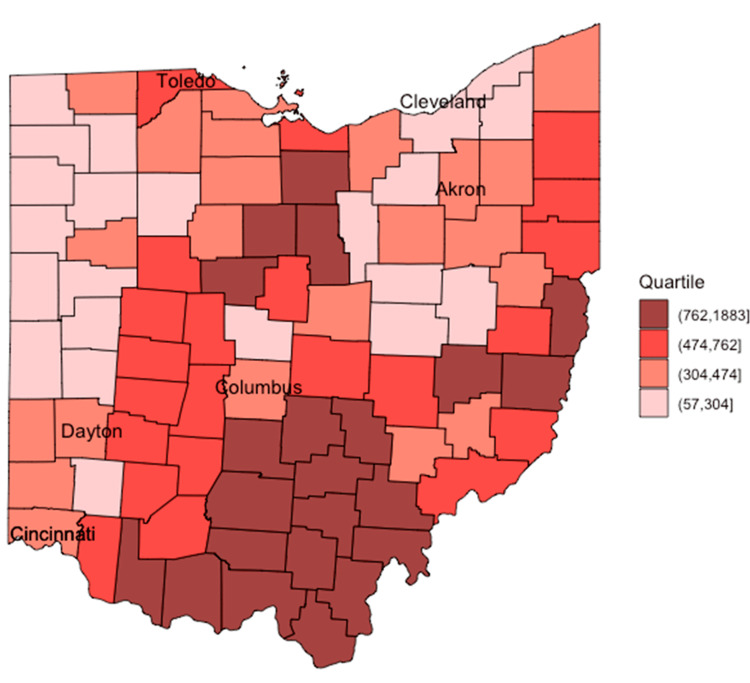
County-level opioid misuse prevalence per 100,000 in 2018

The county-level opioid misuse prevalence per 100,000 in 2018, as estimated by integrated abundance modeling, is mapped in Figure 1. The highest estimated opioid misuse prevalence was primarily observed in the southern and eastern portions of the state.

**Figure 2:**
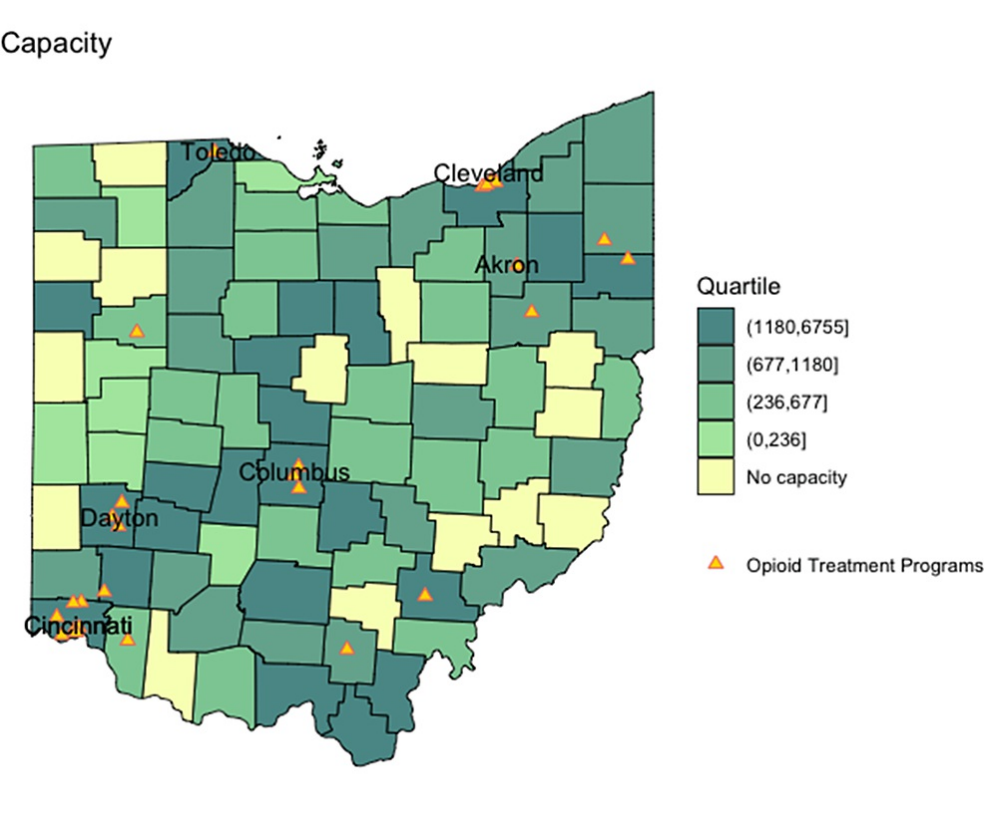
Buprenorphine prescribing capacity per 100,000 in 2018 Estimated number of patients in each county that could be served by office-based buprenorphine

**Figure 3:**
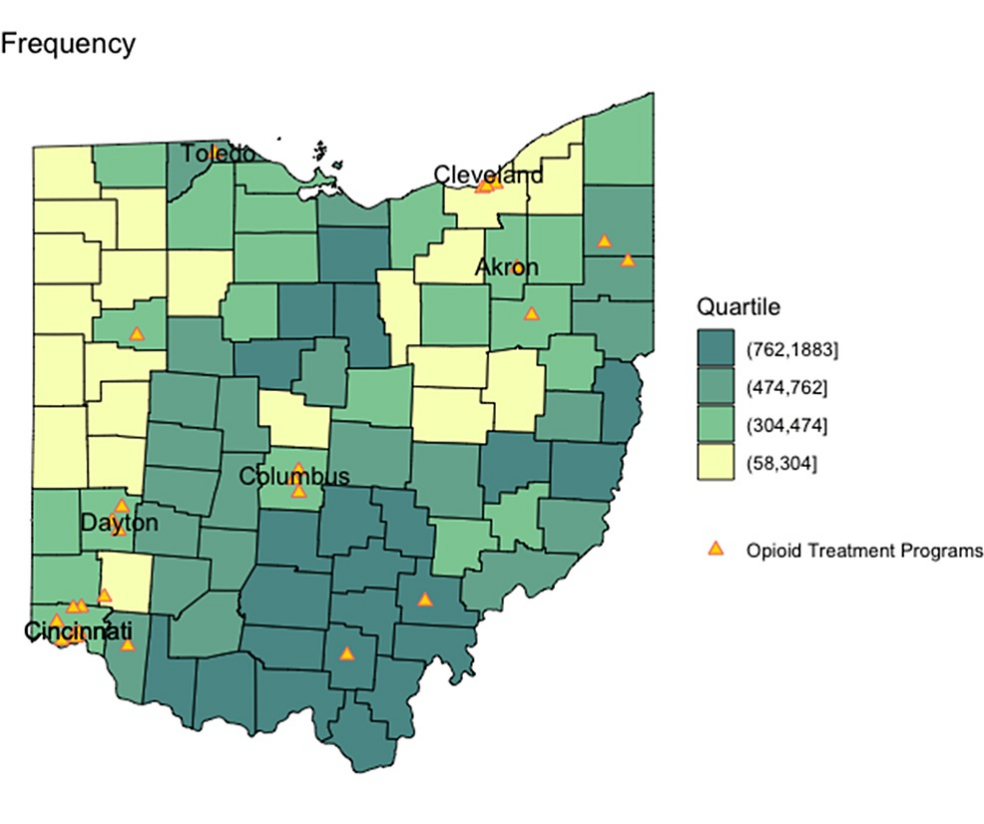
Buprenorphine prescribing frequency per 100,000 in 2018 Number of patients in each county served by office-based buprenorphine

County-level office-based buprenorphine prescribing capacity and frequency per 100,000 are mapped in Figure 2 and Figure 3. Unlike opioid misuse prevalence, buprenorphine prescribing capacity was not predominant in one geographic area. However, counties with the highest buprenorphine prescribing frequency were primarily observed in the southern and eastern portions of the state, similar to opioid misuse prevalence.

**Figure 4:**
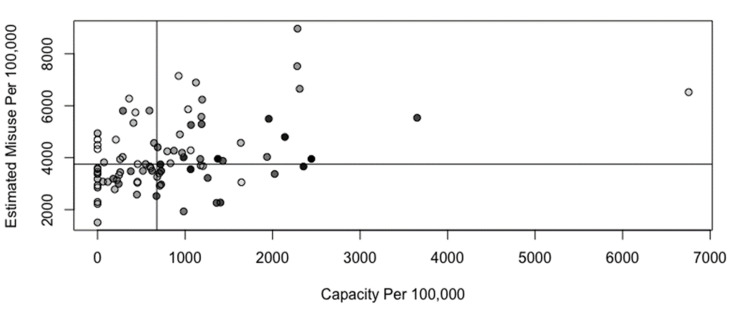
Plot of estimated opioid misuse prevalence per 100,000 versus office-based buprenorphine prescribing capacity per 100,000 Point shading in scatterplot represents county population; darker shading signifies higher population. Reference lines show the state median rates.

**Figure 5:**
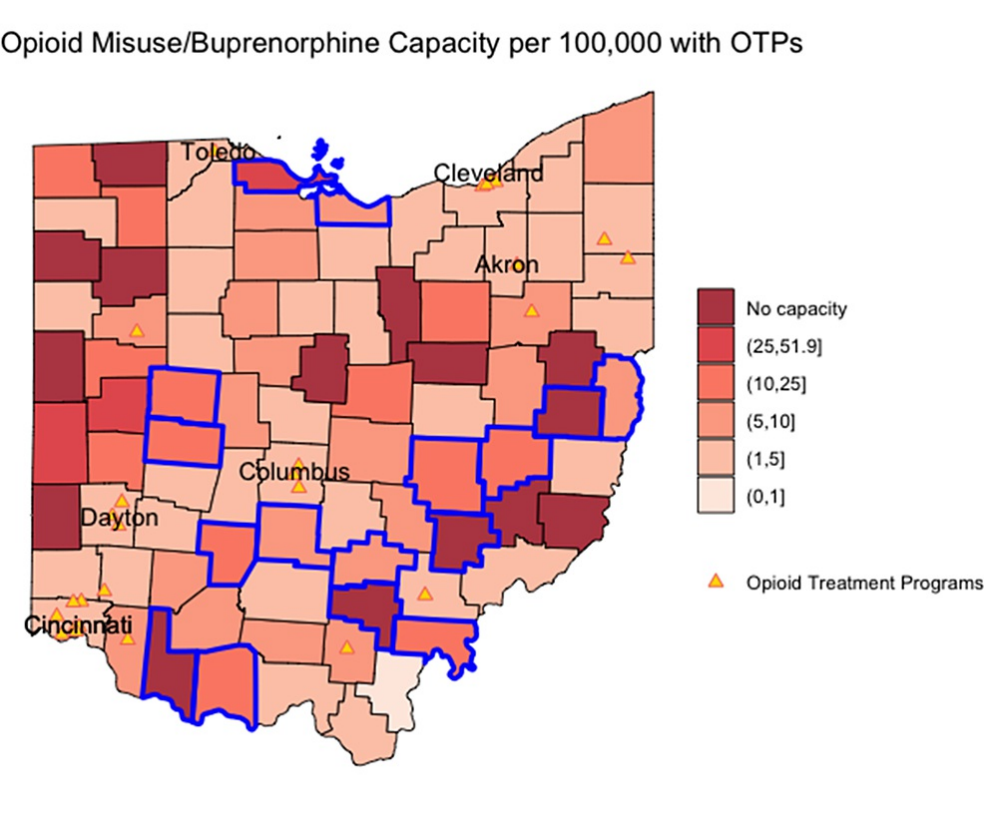
Map of the ratio of opioid misuse prevalence relative to buprenorphine prescribing capacity Blue-bordered counties represent counties where the estimated opioid misuse prevalence is above the statewide median and the buprenorphine prescribing capacity is below the state median. OTP: opioid treatment program

The relationship between estimated opioid misuse prevalence and office-based buprenorphine prescribing capacity is shown in Figure 4 and Figure 5. Reference lines show the state median rates in each scatterplot, and point shading reflects the population size. Counties outlined in blue on the corresponding maps in Figure 4 and Figure 5 represent those where the estimated opioid misuse prevalence was above the statewide median and the buprenorphine prescribing capacity was below the state median. Counties with the highest ratio of estimated opioid misuse relative to buprenorphine prescribing capacity were not predominant in one geographic area.

**Figure 6:**
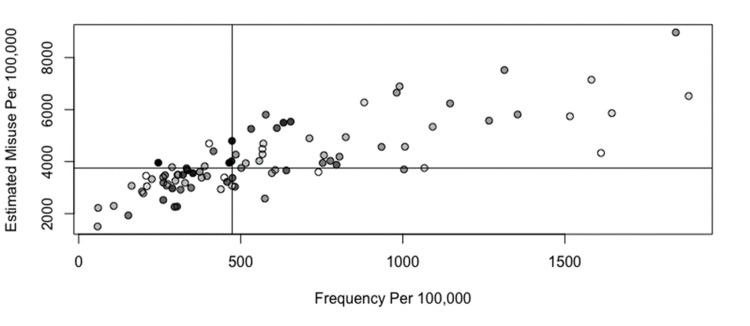
Plot of estimated opioid misuse prevalence per 100,000 versus office-based buprenorphine prescribing frequency per 100,000 Point shading in scatterplot represents county population; darker shading signifies higher population. Reference lines show the state median rates.

**Figure 7:**
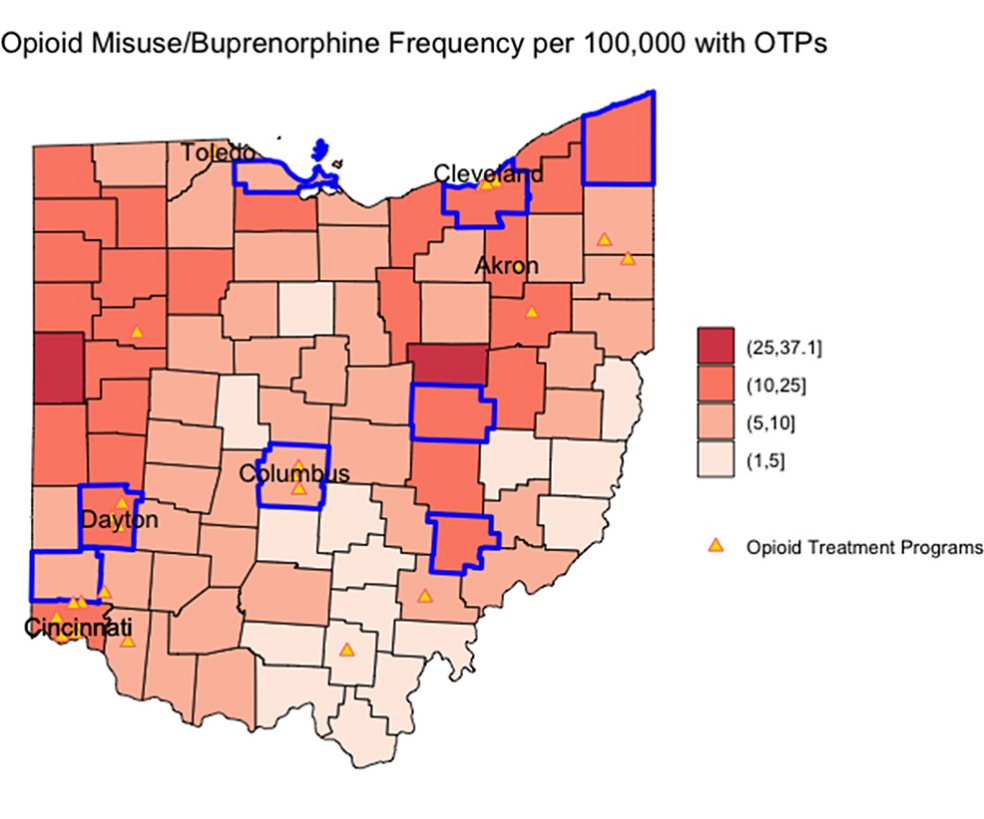
Map of the ratio of opioid misuse prevalence relative to buprenorphine prescribing frequency Blue-bordered counties represent counties where the estimated opioid misuse prevalence is above the statewide median and the buprenorphine prescribing frequency is below the state median. OTP: opioid treatment program

The relationship between estimated opioid misuse prevalence and office-based buprenorphine prescribing frequency is shown in Figure 6 and Figure 7. Reference lines show the state median rates in each scatterplot, and point shading reflects the population size. Counties outlined in blue on the corresponding maps in Figure 6 and Figure 7 represent those where the estimated opioid misuse prevalence was above the statewide median and the buprenorphine prescribing frequency was below the state median. Counties with the highest estimated opioid misuse relative to buprenorphine prescribing frequency were primarily observed in the northern and western portions of the state. The southern and eastern portions of the state had a lower ratio of opioid misuse relative to buprenorphine prescribing frequency.

For both buprenorphine prescribing capacity and frequency, counties in the highest categories demonstrate a higher burden of opioid misuse per office-based buprenorphine access. For example, counties in the highest categories have at least 25 people who are estimated to misuse opioids per each individual able to or receiving treatment, signaling a lack of prescribing access in those counties relative to the estimated potential demand for treatment. Unlike opioid misuse prevalence and buprenorphine prescribing frequency, which were geographically highest in the southern and eastern portions of the state, OTPs mapped predominantly to urban counties with major metropolitan areas.

## Discussion

In this descriptive ecological study, we found that less than half of waivered providers in the state of Ohio in 2018 were prescribing buprenorphine, and 25% of counties had no buprenorphine access. In addition, while opioid misuse prevalence and buprenorphine prescribing frequency demonstrated similar spatial patterns, with highest levels in the southern and eastern portions of the state, office-based buprenorphine prescribing capacity did not. It is important to note that our findings represent a cross-sectional investigation of spatial patterns; changing substance misuse burden and prescribing patterns require real-time analyses of these trends [17].

Buprenorphine access was different in urban versus rural counties. In urban counties, particularly those with major metropolitan areas, buprenorphine prescribing capacity was high, while buprenorphine prescribing frequency was relatively low, suggesting that many waivered providers were not prescribing buprenorphine or were so to a limited number of patients. In contrast, in rural counties, buprenorphine prescribing capacity and frequency were very similar, highlighting the need for additional buprenorphine-prescribing providers in these areas. While additional providers might be helpful in urban areas, unless the gap between capacity and frequency is understood, buprenorphine access may still be limited.

It is worth considering how the recent removal of the DATA 2000 waiver requirement will impact buprenorphine access moving forward. It is postulated that deregulation would improve access to buprenorphine [18]. Whether deregulation would impact buprenorphine prescribing capacity and frequency equally is not known. Buprenorphine training requirements and waiver applications have limited the number of providers who could prescribe buprenorphine [19]. The removal of these requirements could have a substantial impact on buprenorphine prescribing capacity and improve access where capacity is a major barrier to treatment access, such as in rural counties in the current study.

Deregulation may also impact buprenorphine prescribing frequency as regulations reinforce stigma, constraining access and discouraging patient engagement and retention in treatment [19]. Marginalization to prescribe buprenorphine, created by waivers, undermines the principle that opioid use disorder is like other chronic medical or mental health conditions that are managed by primary care clinicians. Removing buprenorphine prescribing regulations in France yielded increases in its use by persons with opioid use disorder and accelerated integration into primary care [20].

Our mapping of OTP locations demonstrated that they were concentrated in counties with major metropolitan areas, and they did not show the spatial pattern seen for opioid misuse prevalence and buprenorphine prescribing frequency. Along with mainstreaming of buprenorphine, there have been calls for mainstreaming of methadone [21]. The National Institute on Drug Abuse Clinical Trials Network recently convened the Methadone Access Research Task Force to develop a research agenda to expand and create more equitable access to methadone treatment for opioid use disorder. The identified areas where research is needed included access to methadone in general medical and other outpatient settings, and optimal educational and support structure for the provision of methadone by medical providers [22].

The limitations of the current study should be considered. As noted above, our findings represent a one-time cross-sectional view of spatial patterns and may not represent current treatment access and opioid misuse burden in the state of Ohio. In addition, for buprenorphine capacity, we assumed that each physician only prescribed office-based buprenorphine in the county in which they registered. This assumption may be incorrect as physicians may list a county other than the one that they work in on their DATA 2000 waiver paperwork or may work in multiple counties. Additionally, these office-based buprenorphine prescribing capacity and frequency data were limited to physician prescribers, which may not reflect more recent access as more advanced practice providers became certified and began prescribing office-based buprenorphine in 2017, especially in rural areas [23].

We also assumed that all treatment admissions or opioid overdose deaths were representative of individuals requiring treatment. While this may be a reasonable assumption, it is unlikely to be universally true, particularly as fentanyl is unknowingly added to other substances [17,24]. As such, our estimate of opioid misuse may overestimate the number of individuals needing treatment. In addition, we assumed that the estimates of the prevalence of opioid misuse reflected a potential upper bound on the demand for treatment but recognize that all people who misuse opioids may not seek treatment even if it were accessible.

## Conclusions

In summary, in the state of Ohio, buprenorphine prescribing frequency shared a similar geographic distribution to opioid misuse prevalence, whereas buprenorphine prescribing capacity did not. Urban counties had higher buprenorphine capacity relative to their burden of opioid misuse; access was limited more by the number of providers who were actually prescribing. In contrast, rural counties had lower buprenorphine capacity relative to their burden of opioid misuse, but a minimal gap was evident between prescribing capacity and frequency, suggesting that access was limited by the number of available providers. The location of OTPs, which were predominantly in counties with major metropolitan areas, added to the complexity of MOUD access. While the recent deregulation of buprenorphine prescribing should help improve buprenorphine access, future research should investigate whether deregulation impacts buprenorphine prescribing capacity and buprenorphine prescribing frequency similarly.
